# Surgical Approaches to Upper Limb Spasticity in Adult Patients: A Literature Review

**DOI:** 10.3389/fresc.2021.709969

**Published:** 2021-08-31

**Authors:** Mahdis Hashemi, Nadine Sturbois-Nachef, Marry Ann Keenan, Paul Winston

**Affiliations:** ^1^Canadian Advances in Neuro-Orthopedics for Spasticity Congress, Victoria, BC, Canada; ^2^Vancouver Island Health Authority, Victoria, BC, Canada; ^3^Orthopédic and Traumatologic Département, University Hospital of Lille, Lille, France; ^4^Neuro-Orthopaedics, MossRehab Hospital, Elkins Park, PA, United States; ^5^Orthopaedic Surgery (Ret), University of Pennsylvania, Philadelphia, PA, United States; ^6^Department of Physical Medicine and Rehabilitation, University of British Colombia, Vancouver, BC, Canada

**Keywords:** spasticity, upper limb, orthopedic surgical procedure, cerebral palsy, stroke, traumatic brain injury, spinal cord injury, multiple sclerosis

## Abstract

**Introduction:** Spasticity is the main complication of many upper motor neuron disorders. Many studies describe neuro-orthopedic surgeries for the correction of joint and limb deformities due to spasticity, though less in the upper extremity. The bulk of care provided to patients with spasticity is provided by rehabilitation clinicians, however, few of the surgical outcomes have been summarized or appraised in the rehabilitation literature.

**Objective:** To review the literature for neuro-orthopedic surgical techniques in the upper limb and evaluate the level of evidence for their efficacy in adult patients with spasticity.

**Method:** Electronic databases of MEDLINE, EMBASE, CINAHL, Cochrane Central Register of Controlled Trials, and Cochrane Database of Systematic Reviews were searched for English, French as well as Farsi languages human studies from 1980 to July 2, 2020. After removing duplicated articles, 2,855 studies were screened and 80 were found to be included based on the criteria. The studies were then divided into two groups, with 40 in each trial and non-trial. The results of the 40 trial articles were summarized in three groups: shoulder, elbow and forearm, and wrist and finger, and each group was subdivided based on the types of intervention.

**Results:** The level of evidence was evaluated by Sackett's approach. There were no randomized control trial studies found. About, 4 studies for shoulder, 8 studies for elbow and forearm, 26 studies for wrist and finger (including 4 for the thumb in palm deformity), and 2 systematic reviews were found. Around, two out of 40 trial articles were published in the rehabilitation journals, one systematic review in Cochrane, and the remaining 38 were published in the surgical journals.

**Conclusion:** Most surgical procedures are complex, consisting of several techniques based on the problems and goals of the patient. This complexity interferes with the evaluation of every single procedure. Heterogenicity of the participants and the absence of clinical trial studies are other factors of not having a single conclusion. This review reveals that almost all the studies suggested good results after the surgery in carefully selected cases with goals of reducing spasticity and improvement in function, pain, hygiene, and appearance. A more unified approach and criteria are needed to facilitate a collaborative, evidence-based, patient referral, and surgical selection pathway.

## Introduction

The term spasticity was first introduced in English by Good in 1829 (1). The definition has undergone continual change with a recent definition by Dressler et al. (2) as “involuntary muscle hyperactivity in the presence of central paresis which can consist of spasticity sensu strictu, of rigidity, of dystonia and of spasm or a mixture of those elements.” The evolving terminology underscores the challenge of categorizing a condition with individualized presentations in which some maintain spasticity with a reducible deformity pattern vs. others that develop musculotendinous retraction or contracture. These manifestations of hypertonicity are the main complication in many upper motor neuron disorders, such as cerebral palsy (CP), traumatic brain injury (TBI), stroke, multiple sclerosis (MS), and spinal cord injury (SCI), with about 40% prevalence in patients after stroke (3) and 65% in patients with SCI (4). Spasticity may lead to stiffness of the affected muscles, joints, and surrounding soft tissue and ultimately contracture (5). Restricted range of motion (ROM) due to spasticity or contracture may cause pain, skin breakdown, and have a significant influence on daily activities including mobility, feeding, hygiene, and dressing. There are many guidelines for managing spasticity with treatments typically divided into two categories: pharmacological, such as oral anti-spasmodic, baclofen pumps, injectable botulinum toxin, and non-pharmacological treatments, including adjunctive treatments such as bracing and physical therapy and surgical techniques (2, 6–9). The burden of medical care and literature provided for patients with spasticity is provided by rehabilitation specialists, however, many rehabilitation focused guidelines do not include surgery in their algorithms, with few publications for surgical management in the rehabilitation literature, despite the literature on surgical correction of limb and joint deformities dating to the early nineteenth century (10).

Spastic deformities result from an imbalance between hyperactive and weakened muscles. The early neuro-orthopedic surgical techniques focused on improving the muscle balance, stabilizing the affected joints, and correcting limb deformity due to prolonged spasticity or contracture. Bankart (11), addressed the efferent pathway of reflex on muscle and nerve in the spastic limb. The surgical techniques are all typically reserved for patients with spasticity that is refractory to more conservative methods. They can be characterized as the more traditional muscle or tendon release or newer surgical techniques such as neurectomy which is the total or partial surgical sectioning of a motor branch reserved for muscles that are believed to be fully reducible deformities upon assessment (6, 9, 12–14). Horstmann et al. (15), in a review of patients with CP who underwent an orthopedic surgery between 1999 and 2005, reported that out of 114 patients with spasticity, 57 patients had a total of 144 upper extremity procedures.

While there are many studies describing neuro-orthopedic procedures and techniques, there is neither any available summary of the evidence of surgical intervention nor the potential surgical approaches in the rehabilitation literature. The upper limb, in addition, has limited literature when compared with the lower limb. There is currently no standard method to assess and select a patient for a given surgical intervention between centers with differing methodologies. To increase the appropriate selection of patients with problematic spasticity, these experts require an understanding of the available procedures and the indications for each. This article will introduce surgical concepts to the rehabilitation audience and review available articles for different surgical approaches to upper limb surgery for the adult patients.

## Objectives

To review the literature for neuro-orthopedic surgical techniques in the upper limb and the quality of evidence for their efficacy in adult patients with spastic acquired neurological disorders.

## Methods

A literature search was performed by the College of Physicians and Surgeons of British Columbia librarians, for all related articles between 1980 and July 2020, using electronic databases of MEDLINE, EMBASE, CINAHL, Cochrane Central Register of Controlled Trials, and Cochrane Database of Systematic Reviews. A sample of search strategy that has been applied for the MEDLINE database is attached as Appendix 1. The search was restricted to the languages of the authors, English, French, and Farsi languages, and was based on the main concepts of spasticity and surgery. Cerebral palsy (CP) was added as an additional search term due to many articles arising from this term being missed by spasticity. The inclusion criteria for studies were as follows:

- Upper limb spasticity due to any acquired reason or CP.
- All articles with participants mean age of more than 18 years.
- Having any type of soft tissue surgery or arthrodesis in the spastic upper limb.

All titles and abstracts of articles were screened for eligibility. The full texts of all the eligible articles were reviewed one more time and any disagreement was discussed; in case of further disagreement, a third person was asked for the resolution. The articles not recognized as eligible were not reviewed. Since no randomized control trials (RCT) were found, articles were categorized into two groups: trial and non-trial. Non-trial articles are used in the text of this manuscript while all trial studies were reassessed for the level of evidence by Sackett's approach (16). This method of evaluation grades the evidence on a well-accepted five-point scale, based on their design. Data were entered in an excel spreadsheet in three groups: (1) shoulder, (2) elbow and forearm, and (3) hand and wrist. Mixed surgical approaches were found within the same article. Each group was categorized into four subgroups based on the predominant intervention performed in the article: tendon/muscle release or lengthening, tendon transfer, neurectomy, and arthrodesis.

## Results

**Figure 1:**
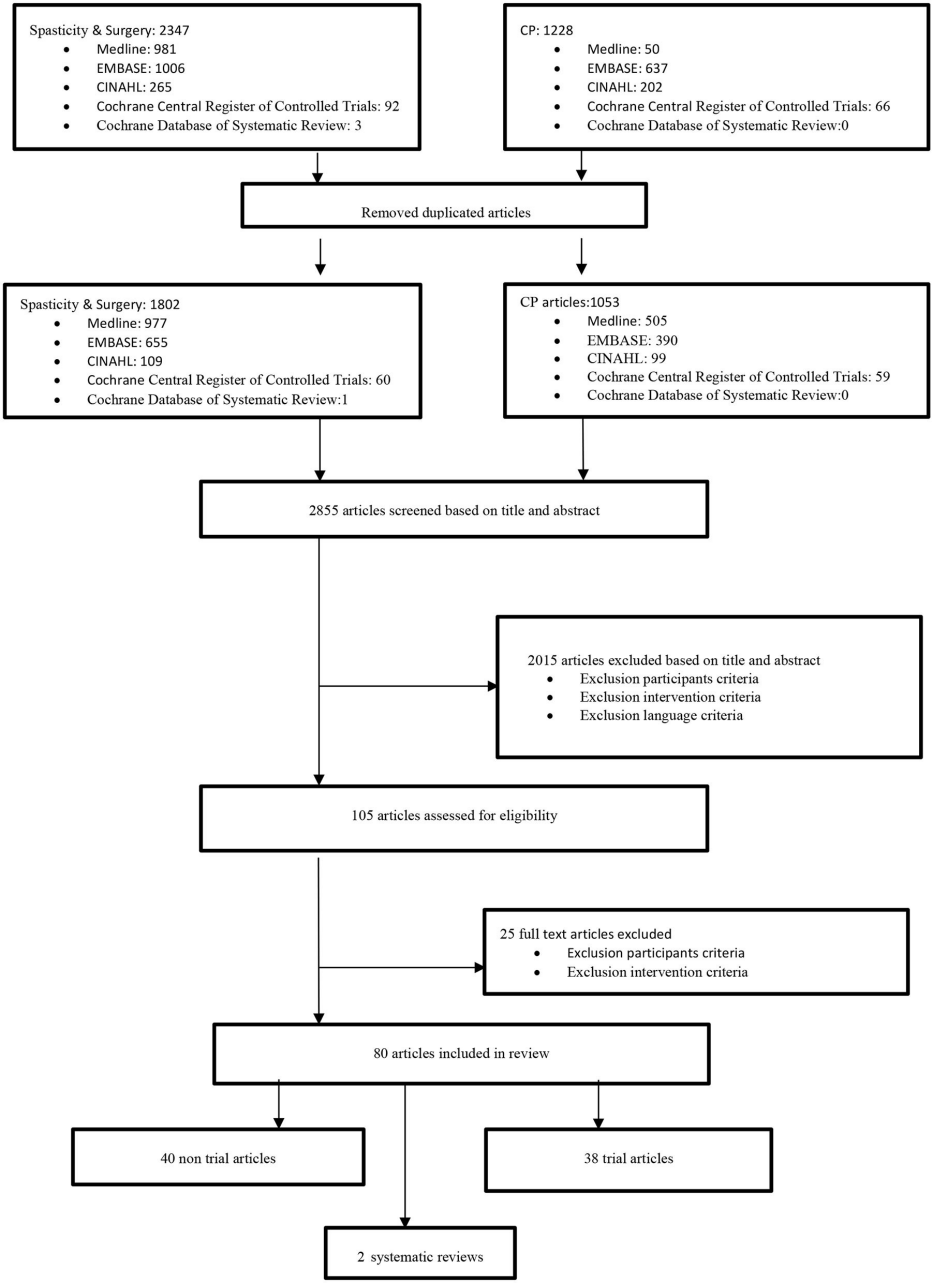
Data collection flowchart.

After removing duplicate articles, 2,855 articles were retrieved for screening based on their titles and abstracts. Based on inclusion criteria, 80 articles were reviewed (40 non-trials, 38 trials, and 2 systematic reviews). Trial articles (including case report studies) contain four studies for shoulder, eight studies for elbow and forearm, and 26 articles for wrist and finger (including four studies for the thumb-in-palm deformity). One systematic review for neurectomy and one other for thumb-in-palm deformity in CP patients were included separately. Only two articles out of 38 trial articles were published in rehabilitation journals, and three were published in general medical journals, while 33 articles were published in surgical journals. Of the two systematic reviews, one was published in Cochrane and the other one in a surgical journal (Figure 1).

### Main Procedures

The literature review revealed heterogeneous techniques and approaches to spastic limb and surgical interventions. The deformities were typically divided into two groups based on the underlying factors and could be most easily grouped into the following: static group, such as fracture malunion, joint subluxation, adhesive capsulitis, heterotopic ossification, and soft tissue contracture, and dynamic group which is the result of neurogenic factors such as weakness, spasticity, rigidity, impaired motor control, and spastic reactions triggered by distant stimuli (17). Patients with spasticity as the main factor of the deformity were often further categorized into two main groups: those with some volitional motion and those without (18). For those without volitional motion, the goals were to facilitate care such as hygiene, comfort, and reduce pain, while the volitional groups were assessed for the potential functional hand (18, 19). Keenan et al. noted that potentially functional hands require intact sensibility, two-point discrimination of 10 mm or less, and volitional motor control of selected joints, while non-functional hands have no motor control and sensibility for function (20). The upper extremity procedures were thus divided into two groups (21). The first group addressed limbs with volitional control, such as fractional lengthening, Z-lengthening, origin release or slide, and joint osteocapsular release. The procedures for limbs without volitional control are muscle origin release (slide), myotomy, tenotomy, neurotomy, arthrodesis, and joint osteocapsular release. Non-functional upper extremities underwent muscle origin release, myotomy, and tenotomy indicated for muscles with severe spasticity or contracture (static deformity). Neurectomies were used in potentially functional limbs to retain some activity while neurotomy or total neurectomy (complete sectioning of a nerve trunk) were recommended to be used in the non-functional limbs with severe spasticity to facilitate hygiene, nursing, and to improve cosmesis (19).

### Shoulder

The paretic shoulder is a common source of pain in patients with upper motor neuron disorders and can impede care. The spasticity most commonly reveals a flexion pattern in the upper limb (20) with adduction and internal rotation of the shoulder as a result of unbalanced spasticity of internal rotators *(latissimus dorsi, teres major, pectoralis major, and subscapularis*) (22).

Several procedures are described to address the flexed adducted shoulder. We found two case series for tenotomy and muscle lengthening procedures and two case series for neurectomy. No RCT were found (Table 1).

**Table 1:** Summary of articles for surgical approaches to a spastic shoulder.

**References**	**Level of evidence**	**Type of study**	**Number of cases**	**Follow up**	**Performed interventions**	**Result**
**Shoulder tendon lengthening/Muscle release**						
Namdari et al. (23)	V	Case series	34	12.2 months	Shoulder tendon fractional lengthening *(pectoralis major, latissimus dorsi, teres major)*	- MAS improved from 2.4 preoperatively to 1.9 postoperatively - Improvement in active flexion, abduction, and external rotation in comparison to the normal contralateral side with most dramatic gains in external rotation - Passive extension, flexion, abduction, and external rotation improved in comparison to the normal contralateral side - 94% pain improvement - 88% pain free - 92% satisfaction
Namdari et al. (24)	V	Case series	36	14.3 months	Shoulder tenotomies of the *pectoralis major, latissimus dorsi, teres major*, and *subscapularis*	- Passive extension, flexion, abduction, and external rotation improved from 50, 27, 27, 1% to 85, 70, 66, and 56%, respectively - 100% pain improvement - 95% pain free - 97% satisfaction with surgery - All patients reported improved axillary hygiene and skin care.
**Neurotomy**						
Sitthinamsuwan et al. (25)	V	Case series	Total of 141. 14 pectoral neurotomy	Before and after	Lateral pectoral nerve branches selective neurotomy	MAS: Mean of 2.6 improvement PROM: 20.6° improvement
Decq et al. (26)	V	Case series	5	11 months	−5 neurotomies of *pectoralis major* (all patients) and 2 neurotomies of *teres major* - All of them benefited from associated neurotomies for elbow, wrist and fingers, through the same operation or later	- Improvement in active amplitude: • Abduction (30°) • Antepulsion (50°) • Retropulsion (20°) • External rotation (20°) - Improvement of functionally useful active amplitude score from 2.66 to 5.16 out of 6 (including: standing position, walking stability, improvement in lower limb ROM)

*MAS, modified Ashworth scale; PROM, passive range of motion; ROM, range of motion*.

Tenotomy of the *pectoralis major, latissimus dorsi, teres major, and subscapularis* can be utilized in patients with non-functional upper limb without any voluntary control or with severe contracture to improve the position of the arm (27). A 2011 study reported that 36 patients who had tenotomy and at 14.3 months follow-up, passive extension, flexion, abduction, and external rotation improved significantly and 95% of the participants were pain-free after surgery (24).

Patients who have some voluntary movements are evaluated to identify if the contributing factors are muscle weakness and contracture or inappropriate activation of the antagonist muscles (27). If movement is restricted by the antagonist muscles, the muscle can be selectively lengthened. A study in 2012 reported 34 patients who had fractional lengthening of the pectoralis major, latissimus dorsi, or teres major. In 12.2 months follow-up, significant improvement in passive, active ROM, and pain while 88% rate of being pain-free is reported (23).

Selective peripheral neurectomy (SPN) is used for focal or segmental spasticity. There is scarce literature for SPN in shoulder spasticity. In a retrospective study in 2013 (25), 141 SPN comprising of 14 pectoral, 15 musculocutaneous, 33 medians, and 24 ulnar (55 lower limbs) neurectomies were reported, while the patients who needed concurrent orthopedic surgeries were not included. Most of the patients had non-functional upper limbs, so a small number of patients showed an improvement in function. Since multiple SPN procedures were performed for each patient, analysis of the effects of every single procedure is not possible, however, an increase in the comfort of caregivers was shown in all the cases. In the shoulder, a significant average of 2.6-points improvement on the Modified Ashworth scale (MAS) and 20.6° improvement in passive ROM was reported. The authors suggest that SPN is beneficial in the reduction of spasticity, amelioration of functional status, facilitation of patient care, and the prevention of long-term musculoskeletal sequelae.

### Elbow and Forearm

The upper extremity flexion synergy pattern is common after upper motor neuron (UMN) injury (28). Elbow flexion is the most common posture for spastic elbow and can interfere with daily activity (e.g., clothing and functional use), can be painful, and lead to skin breakdown. In the present review, we did not find any RCT, but there are five studies (four case series and one case-control) for muscle release and tendon lengthening and three case series in neurectomy (Table 2).

**Table 2:** Summary of articles for surgical approaches to a spastic elbow.

**References**	**Level of evidence**	**Type of study**	**Number of cases**	**Follow up**	**Performed interventions**	**Results**
**Tendon lengthening/Muscle release**						
Sharan and Rajkumar (29)	V	Case series	120	Baseline, 5, 12 months	Orthopedic selective spasticity surgery including; intramuscular lengthening and sliding lengthening of elbow flexors, forearm flexors, pronators, hand intrinsic muscles	- Significant improvement in MAS, MACS, MAUULLF - In 55% of the participants with long term follow up, improvement maintained in more than 80% of them in 1 year follow up
Namdari et al. (28)	V	Case series	29	1.7 years	Elbow flexors release (*Biceps brachii, Brachialis, brachioradialis*)	−94% of 17 patients were pain free - Improved passive elbow extension from −78 to −17° - No change in passive elbow flexion - Improvement of MAS from 3.3 (22) to 1.4
Keenan et al. (30)	V	Case series (30)	21	29 months	Proximal release of the *brachioradialis*, Z-lengthening of the *biceps brachii* tendon, myotendinous lengthening of the *brachialis*	- Improvement in elbow arc motion from 62° preoperatively to 111° postoperatively - Improvement in time require (31) d for elbow flexion (2.9 second in comparison to 1.7) - Improvement in time required for elbow extension (4.8 s in comparison to 2.2) - Smoothing of extension pattern - Improvement in upper extremity function in 20 cases
Anakwenze et al. (31)	V	Case series	42	14 months	Fractional elbow flexors lengthening	- Improvement in active flexion (119 to 133°) - Improvement in active extension (42 to 20°) - Improvement in total active arc (77 to 130°) - Improvement in passive flexion (127 to 139°) - Improvement in passive extension (24 to 8°) - Improvement in total passive arc (103 to 131°) - Improvement in MAS (2.7 to 1.9)
Gong et al. (32)	IV	Retrospective case control	Group1: 14. Group 2: 15	Group 1: 72 months Group 2: 31 months	Group1: anterior elbow release including *lacertus fibrosus* division, *brachialis* fractional lengthening and denuding of the *pretendinous adventia* off the *biceps brachii* tendon Group 2: additional partial *biceps brachii* tendon lengthening	- Group 2: more improvement in: • Flexion posture (53° vs. 44°) • Active extension (23° vs. 15°) • Decrease in active flexion (7°) whereas the first group had no change - No differences in forearm supination and House score between 2 groups
**Neurectomy**						
Maarrawi et al. (33)	V	Case series	64 neurectomies in 31 patients: -Musculo - cutaneous nerve:15 -Median nerve:25 -Ulnar nerve: 24	Before and after/mean long term follow up of 4.5 years	Selective peripheral neurotomy	- Improvement of AS from 3.6 ± 0.5 to 0.8 ± 0.77 - Improvement in distal spasticity in 7 out of 15 (46%), without reaching statistical significance - Disappearance of dynamic spasticity all 6 patients who had that preoperatively - Distant effect: decreased spasticity in proximal muscles of the elbow in isolated median and/or ulnar nerve neurotomy (from 2.25 ± 0.86 to 0.94 ± 0.7) - Decreased forearm pronation with median and/or ulnar neurectomy (from 3.24 ± 0.66 to 0.6 ± 0.58) - The mean degree of satisfaction based on VAS (61.5 ± 24.6) - Improvement in function and comfort goals
Sitthinamsuwan et al. (25)	V	Case series	15 out of 141 cases	Before/ after	Musculocutaneous (19) neurotomy	- MAS improvement from 3.2 ± 0.4 to 0.6 ± 0.7 - PROM improvement from 74.7 ± 29.5° to 95.3 ± 17.1°
Leclercq (19)	V	Case series	133 neurectomy in 47 cases (22 adults and 25 children)	15.2 months for adults	Hyperselective neurectomies in different muscles of upper limb and 31 muscle lengthening, 3 tendon transfer and 1 midcarpal arthrodesis	Elbow flexors in 16 months follow up: - Improvement in spontaneous posture (20%) - Increase in antagonist strength (0.9) - No decrease in flexion strength - Limited improvement in AROM (22°) - A decrease in MAS (from 2.8 to 0.7) - High satisfaction in final follow up (average 8.8)

*AROM, active range of motion; AS, Ashworth scale; MAS, Modified Ashworth Scale; MACS, manual ability classification system; MAUULF, Melbourne Assessment of Unilateral Upper Limb Function; PROM, passive range of motion; VAS, visual analog scale*.

Namdari et al. (28) performed elbow flexors releases (*brachialis, brachioradialis, biceps brachii*) in patients with no active volitional control of the upper extremity to improve passive motion and pain related to the elbow flexion spasticity. Their results show that 16 out of 17 (94%) patients were completely pain-free in a mean follow-up of 1.7 years after the procedure. MAS decreased to 1.4 from 3.3 and passive elbow extension improved from a lack of 78 to 17°, while passive elbow flexion remained almost the same (141° in comparison to 143°). All patients showed improvement in the upper body dressing and antecubital hygiene.

Muscle or tendon lengthening for the functional upper limb was described by Keenan et al. (30). They performed modified elbow flexor release which consisted of proximal release of brachioradialis, Z-lengthening of the biceps tendon, and myotendinous lengthening of *brachialis* muscles. Twenty-one patients with spasticity secondary to traumatic brain injury (TBI) with a mean follow-up of 29 months were included. There was an improvement in elbow arc motion from 62° preoperatively to 111° postoperatively. In addition, the study identified a significant improvement in the time required for elbow flexion (2.9 s in comparison to 1.7 s) and the time for elbow extension (4.8 s in comparison to 2.2 s) with smoothing of extension pattern. The improved elbow motion resulted in upper extremity function for 20 out of 21 patients. In 2013, Anakwenze et al. (31) performed myotendinous lengthening of elbow flexors in 42 patients with multiple etiologies, most commonly stroke, who all had a functional upper limb preoperatively. The significant improvement in active extension, active and passive arc motion, and MAS was demonstrated.

Gong et al. (32) investigated the effect of adding a partial *biceps brachii* lengthening procedure to anterior elbow release in a retrospective case-control study. They compared two groups of patients with an elbow flexion contracture of more than 50° who had received anterior elbow release (*lacertus fibrosis* division, *brachialis* fractional lengthening, and denuding of the *pretendinous adventia* off the *biceps brachii* tendon), and a second group who had additional *biceps* lengthening. Both the groups consisted of adults and children but the mean age for the groups was 21 and 20 years, respectively. Mean follow-ups were 72 and 31 months, respectively. The achieved result showed that adding this procedure may improve elbow flexion posture (53° vs. 44°) and active elbow extension (23° vs. 15°). They found no differences in the forearm supination or House score between the two groups.

Maarrawi et al. (33) in a cohort study reported 64 cases of SPN in 31 patients comprising of 15 musculocutaneous, 25 medians, and 24 ulnar neurotomies. The patients were re-examined at 2, 6, and 12 months after surgery and the long-term evaluation with a mean of 4.5 years. They showed the MAS improved from 3.6 ± 0.5 preoperatively to 0.8 ± 0.77 postoperatively. The patients who had an isolated median and/or ulnar nerve neurotomy observed a significant decrease in spasticity in what was termed the “proximal muscles of elbow.” Dynamic spasticity (severe elbow flexion induced by walking) and gait improvement in 6 out of 15 patients with musculocutaneous neurotomy were significant.

Leclercq (19), in a prospective study, reported the results of hyper selective neurectomy (HSN) in 47 patients (22 adults and 15 children with average age of 33 years). An average of 2.8 neurectomies was performed. Other performed procedures were muscle lengthening, tendon transfer, and midcarpal arthrodesis. In long-term follow-up, the result showed a significant improvement in elbow flexors spontaneous posture (60%), antagonist strength (0.9 points) without a decrease in the flexion strength. While a decrease in spasticity and satisfaction at final goals were significant (1.8 and 8.8), the active ROM improvement was limited (22°). It was concluded that this technique is promising in reducing spasticity, improvement of ROM, and without any loss of strength. In both the studies, multiple neurectomies and other orthopedic procedures have been done for each case, so the improvements are not purely the result of neurectomy.

### Wrist and Finger

We identified 26 articles: six case series and one case-control study in tendon lengthening/release, four case series and one case-control study in neurectomy, six case series in tendon transfer, four case series in arthrodesis, and three case series and 1 case-control study in thumb-in-palm deformity (Supplementary Table 1).

The most common flexion pattern presents as a flexed wrist with a clenched fist which is the result of the overactivity of flexor muscles. Keenan et al. (20) reported the result of finger flexors fractional lengthening in 27 patients. They divided patients into two groups of potentially functional hands and non-functional hands. Each patient had at least three finger flexors lengthened. Five patients had non-functional hands preoperatively, and they showed an improvement in appearance, posture, and hygiene. In the second group with 22 patients who had some motor control and intact sensibility preoperatively, hand function increased in 20 patients (91%) and 17 patients (77%) revealed an increase in the use of a whole upper extremity, while 4 (19%) remained unchanged. The decrease in function due to overlengthening of flexor muscles and loss of grip is reported in one patient. They reported fractional lengthening as a technically simple procedure. The potential errors were reported as insufficient lengthening in non-functional hands and overlengthening in functional hands which may cause persistent flexion deformity and loss of grip, respectively.

In a heterogeneous prospective cohort study, Bergfeldt et al. (34) reported 30 patients who had tendon lengthening and muscle release for improving extension and supination. A mean of 1.4 improvements in MAS and significant improvement in resting position, improvement of the wrist, finger, and thumb passive ROM and active were reported. Wangdell et al. (5) reported the result of the procedure of tendon lengthening for wrist flexors and finger flexors combined with the additional procedure of pronator teres, thumb adductor, and intrinsic tendon release if necessary, in tetraplegic, SCI patients. The most common tendons lengthened were *flexor digitorum superficialis* (FDS). They reported significant general improvement in both performance and satisfaction with the largest improvements made during the first 6 months which were relatively stable between 6 and 12 months of follow-up.

Tenotomy and muscle release are procedures that mostly are considered for patients with a non-functional hand. Thevenin-Lemoine et al. (35) in a retrospective review reported the results of the Page-Scaglietti technique to release *flexor digitorum profundus* and *superficialis* (FDP and FDS), *flexor carpi radialis* (FCR), and *flexor pollicis longus* (FPL), and Z-lengthening of *flexor carpi ulnaris* (FCU) if necessary, in 54 hands. If the wrist extensors were not able to extend the wrist to neutral, tenodesis of *extensor carpi radialis brevis* (ECRB) or a transfer of FCU to ERCB were done. During 26 ± 21 months follow-up, wrist extension improvement was significant both with fingers flexed and extend and all patients agreed that their treatment goals had been achieved. This surgery unmasked hidden spasticity/contracture of intrinsic muscles in seven cases. Patients with spasticity of intrinsic muscles may benefit from the excision of digital extensor hood as investigated by Reinholdt and Fridén (36). They divided participants into two groups of mild, with focal spasticity, and severe, in which the intrinsic spasticity was hidden behind spastic superficialis and deep flexor muscles and became obvious after transferring or lengthening of extrinsic muscles. In this study, they modified the original method to triangular resect of the ulnar side of dorsal aponeurosis (both lateral band and oblique fibers). Both groups received the same postoperative active training and a resting splint with MCP, and the PIP joints extended, between sessions and after 4 weeks, they started the task-oriented exercise. ROM increased in both the groups; however, patients in the severe group needed longer to show improvement (~3 months) in comparison to the mild group (1 month). Saintyves et al. (37) described the results of a complex of surgeries for hand deformity due to intrinsic muscles spasticity, such as ulnar selective neurectomy in four hands (reduced diameter to 4/5), tenotomy of interosseous muscles in 54, tenotomy of the *abductor digiti minimi* in 18, and metacarpal disinsertion of the interosseous muscles in 6 cases. The hand was immobilized in extension at the MCP for 45 days. Of a total of 67 hands operated, 63 had good results as defined in their primary contracts, such as hygiene, analgesia, and aesthesis, with four relapses.

Many articles suggest tendon transfer to enhance function or to ease the care in spastic hands. Transfer of the FDS tendon to the FDP is one of the most common techniques (38–41). Keenan et al. (38) reported full accomplishment of goals after the procedure in 34 clenched-fist which were hand open position with the resolution of hygienic problems of palmar skin.

Pinzur et al. (42) described less common tendon transfers, which included *brachioradialis* to finger extensor tendon transfer in four patients. All hands had volitional control of wrist extension, overpowered by spastic wrist, and finger flexors with no volitional control of finger extensors. The results indicated good assistive prehensile functional capacity.

Wrist flexors tendon transfer to the radial wrist extensors such as FCU to *extensor carpi radialis* is a procedure indicated in the dynamic wrist deformities which have no static contracture and may have some extension but usually to 45° less than neutral (43).

SPN may be useful when spasticity is localized to muscles that are innervated by one or few peripheral nerves (44). To achieve the best outcome, the target nerve should be selected carefully. There are several articles describing neurectomy of the median nerve to the wrist or the ulnar nerve to control intrinsic muscles spasticity (25, 33, 44, 45). Fouad (44) reported 10 patients with spastic hyperflexion of wrist and fingers who underwent median and ulnar SPN and depending on the degree of preoperative spasticity, 50–80% of the isolated motor branches of fascicles were resected. At a mean follow-up of 21 months, recurrence due to insufficient sectioning was reported in one case (10%), 40% had more than three grades improvement in MAS, 40% had two grades of improvement in MAS, 10% had one grade, and 10% had no improvement. The improvement in abnormal hand posture reported in 90% of patients and all participants who had pain preoperatively (50% of all cases) showed improvement as measured by visual analog scale (VAS).

Wrist fusion or proximal row carpectomy is performed in the skeletally matured patient with severe wrist joint contracture (>45°) limiting functional use of the hand (43, 46). Van Heest and Strothman (47) reported 41 wrists with severe spastic flexion who were treated with wrist arthrodesis using a dorsal approach. The Disability Assessment Scale scores (10 worst, 0 best) improved from 9.6 to 5.5, and VAS (0 much worse, 10 much better) revealed improvement in appearance (7.9), function (6.0), ease of daily care (7.0), and hygiene (6.2). In the study, 94% of patients were satisfied with an average satisfaction VAS score of 8.3. The same procedure was studied by Hargreaves et al. (48) for spastic, flaccid, quadriplegic, and athetoid CP patients to stabilize the wrist.

Thumb-in-palm (TIP) deformity can interfere with hygiene and function as there is a lack of opposing function to the rest of the fingers for pinching or grasping. Surgical correction follows the sample principles, such as release or lengthen the spastic or contracted muscles, augment the weak or flaccid muscles, and stabilize the joint for severe joint instability or joint contracture (49). Botte et al. (50) included 27 traumatic brain-injured patients in a study to review the TIP deformity surgical management results. Different procedures, such as FPL tendon lengthening, Z-lengthening, and fractional lengthening at the musculotendinous junction, the release of first dorsal interosseous, arthrodesis of the thumb interphalangeal joint, and Z-plasty of the thumb web space were applied. The results were satisfactory in 23 patients. All patients who had volitional control before the operation were functionally improved; however, the pinch and grasp remained weak. In the three patients who had arthrodesis of the IP joint, the pulp-to-pulp pinch was restored, and a useful grasp was obtained. Smith (51) offered FPL tendon transfer for TIP deformity due to spasticity of FPL. In this procedure, the FPL tendon was transferred to the radial side of the proximal phalanx and the interphalangeal joint was stabilized in 15 degrees of flexion by tenodesis or arthrodesis. The study showed improvement in thumb balance and function.

Hidden TIP can be revealed after superficialis to profundus (STP) tendon transfer for correction of clench-fist. Pappas et al. (45) recommended median nerve recurrent branch neurectomy for median innervated intrinsic thenar muscles to be added to ulnar motor nerve neurectomy at the time of STP to prevent TIP.

### Systematic Reviews

Smeulders et al. (52) published a systematic review for the thumb in palm deformity in patients with CP. They found no clinical trials or controlled clinical trials. Nine prospective studies with pre- and post-procedure assessments and 24 different interventions or combinations of them were described. While generally thumb-in-palm surgery was considered satisfactory for both the patients and surgeons in all studies, because of the methodological quality of studies, it was impossible to provide a reliable judgment about the role of surgery for the treatment of this deformity.

Yong et al. (53) in a systematic review investigated the role of SPN in the upper limb. They found seven case series with a total of 174 patients, but because of baseline heterogenicity, a meta-analysis was not possible. They concluded that these procedures appeared to be useful in selected cases, but no firm conclusion can be achieved regarding the best surgical technique or the extent of functional improvement.

## Discussion

Although the neuro-orthopedic surgical procedure for the upper limb was introduced many years ago, there is no single approach. Most of the surgical procedures are complex of several techniques based on the presenting problems of patients, the potential for function, and desired goals. The patient population may be from a myriad of acquired spastic disorders. Due to the evolving terminology of spasticity as a spastic muscle overactivity disorder that may include contracture, the ability to find a consistent search term to perform the literature proved challenging as numerous search strategies do not pick up many of the referenced papers initially. Some authors, such as the study by Gatin et al. (54) classified all of these surgeries as soft tissue surgeries. Other spasticity surgeries include concurrent bone and joint procedures.

A precise preoperative assessment is critical to achieving the best result. Many studies did not include their selection process. More recently, the triage pathway has been described comprising of a detailed clinical evaluation and laboratory assessment, such as diagnostic nerve block, trials of botulinum toxin injection, and dynamic electromyography (17, 19, 28, 35). The pre-surgical assessment is beyond the scope of this article; although the importance of this concept should be acknowledged, described, and standardized as future studies are designed. This will allow for the better assessment of outcomes and comparison of surgical techniques.

The approach to surgery as well as the goals varied widely. With classifications, such as static and dynamic, volitional and non-volitional, and reducible and non-reducible, challenge the ability for a rehabilitation clinician to adequately chose the appropriate patient and will depend on their knowledge of these concepts and their local surgical team.

Many studies emphasized the need for rehabilitation after surgery in their protocols (23, 24, 34), however, there was no consensus on timing or a specific protocol.

Not including complications of these surgical techniques is one of the most significant limitations of many reviewed studies. Complications, such as recurrency (19, 28, 33), limited improvement (55), weakness (20), increasing the severity of TIP deformity, non-union and fracture after wrist arthrodesis (47, 56), overcorrection (50), and wound complications (24, 25), are reported in several studies. The complications varied based on the enrolled patients and applied procedures and analysis of them is beyond the scope of this study.

Most of the studies report the outcomes of a combination of different surgeries. While the purpose of this review is not to evaluate every single procedure, this complexity along with the heterogenicity of participants and the absence of clinical trial studies make any overall conclusions on the level of evidence for different surgical techniques impractical.

We implemented Sackett's evaluation method to grade the existing literature which cannot eliminate publication bias of the treating physicians. All studies along with case reports with a lower level of evidence and those that might have bias are reviewed in this study due to key surgical techniques and results that they offer and the scarcity of trial studies that assess the techniques.

## Conclusion

Specific conclusions about the efficacy of the surgery cannot be made based on the heterogeneity of subjects and procedures. This scoping review illustrates that surgery is one potential component of rehabilitation for patients with upper limb spasticity. In addition to other elements, such as splinting, physical therapy, and pharmacological treatment, surgery can lead to good results in carefully selected cases with the goals of reducing spasticity and improving function, pain, hygiene, and appearance. Non-surgical clinicians specializing in spasticity could benefit from understanding the surgical options available. Different functional outcome measurement tools in the reviewed studies render any analysis impractical.

Most of the included studies have a low level of evidence. Further studies with larger sample size and better study designs comprising of the case-control studies or for more complicated techniques, single-case experimental designed studies are needed. In addition, a consensus over inclusion criteria of a patient, functional outcome measurement tools, and rehabilitation protocol after the procedure could make the comparison between different surgical techniques easier. A more unified approach and criteria are needed to facilitate a collaborative, evidence-based, patient referral, and surgical selection pathway.

## Author Contributions

MH was responsible for the literature review, assignment of evidence, and manuscript preparation. PW was responsible for literature review and manuscript preparation. NS-N and MK were content experts responsible for the appraisal of the literature, interpretation of surgical studies, and manuscript reviews. All authors contributed to the article and approved the submitted version.

## Conflict of Interest

The authors declare that the research was conducted in the absence of any commercial or financial relationships that could be construed as a potential conflict of interest.

## Publisher's Note

All claims expressed in this article are solely those of the authors and do not necessarily represent those of their affiliated organizations, or those of the publisher, the editors and the reviewers. Any product that may be evaluated in this article, or claim that may be made by its manufacturer, is not guaranteed or endorsed by the publisher.
