# Supplementary material for: Surgical Approaches to Upper Limb Spasticity in Adult Patients: A Literature Review
Source: Front Rehabil Sci. 2021 Aug 31;2:709969. doi: 10.3389/fresc.2021.709969 (PMC9397894; doi:10.3389/fresc.2021.709969)
Supplement: Supplementary file 1 [file Table_1.docx]

***Supplementary Table 1/ Summary of articles for surgical approaches to a spastic wrist and/or spastic fingers***

| **Refrences** | **Level of evidence** | **Type of Study** | **Number of cases** | **Follow up** | **Performed Interventions** | **Results** |
| --- | --- | --- | --- | --- | --- | --- |
| **Tendon lengthening/Muscle release** | | | | | | |
| Keenan et al.1987  (20) | V | Retrospective review, Case series | 27 | 33months | -Fractional lengthening of FDS and FDP  - Z-lengthening of FCR and FCU in presence of wrist flexion deformity | -5 Non-functional hands: Improvement in hygiene, appearance and posture  -Functional hands:   - Hand function increased in 91%. (a mean of 3.7 improvement in spastic hand function score) - Improvement in upper extremity use in 77% - 19% remained the same |
| Bergfeldt et al.2020  (34) | V | Case series | 30 | Before, 3, 6, 12months | -Z-lengthening followed by reattachment in lengthened position  - Fractional lengthening of *brachioradialis*  -Muscle release of *pronator teres*/ or *adductor pollicis* when necessary | - -MAS improvement: 1.4 - -Pain reduction of 1.3 (VAS) - -Arm-hand function improvement (n = 30): 2.1 points - -Grip strength improvement (n=18): 4.1 kg - -Key-pinch strength improvement: (n=18): 1.6 kg - -COPM performance improvement (n=13): 3.4 points - -COPM satisfaction improvement (n=13): 3.6 points -Grip and release test improvement (n=9) :43 |
| Wangdell et al. 2018 (5) | V | Case series | 30 | - Before, 6, 12 months | - -Wrist and finger flexors lengthening - -Additional *pronator teres*, thumb adductor & intrinsic release | - -Significance improvement in satisfaction/ performance - -The largest improvement occurred in interpersonal activities like handshaking & holding a child followed by wheelchair propulsion, doing household tasks, personal care, walking |
| Thevenin-Lemoine et al.2013  (35) | **V** | Case series | 54 | 26±21 months | Page-Scaglietti technique | - -Improvement in mean of wrist extension: - 67°±25° With fingers extended - 39°±28° with fingers flexed - -10 non-functional hand became functional as a supportive hand - -Zancolli and House classification - scores increased significantly - - Recurrence of deformity in 12 cases - - Unmasked hidden spasticity and contracture of intrinsic muscle in 7 cases |
| Reinholdt  Fridén 2011 (36) | **III** | Case control | 17 patients in 2 groups:  - Mild: 14 fingers in 7 patients  -Severe: 23 fingers in 10 patients | - 1,3,6 months | - Distal ulnar intrinsic release | - -Mild spasticity: ROM improvement in long finger from 65° to 90° - -Severe spasticity: ROM improvement 35° and 45° for long and ring fingers - -More pronounced immediate effect at 1 month in mild group - -Sever group needed longer time to improve - - COPM score improvement in 10 patients (3.9 points on performance and 4.5 on satisfaction) |
| Saintyves et al. 2011 (37) | **V** | Case series | 56 patients, 67 hands | - 2, 7 years | - -4 ulnar neurectomy - -54 tenotomies of interosseous - -18 tenotomy of *abductor digiti minimi* - -6 metacarpal disinsertion of interosseous muscles | - -63 cases had good results as defined in their primary contract. (aesthetic, hygienic, analgesic in 15 cases, hygienic, analgesic in 32 cases, functional in 21 cases) - -4 cases relapsed |
| Gatin et al. 2017 (54) | **V** | Case series | 70 | - Mean of 6.2 months | Soft tissue surgeries including;  -Tenotomy and intramuscular Z-lengthening of wrist flexors, interosseous, adductor pollicis  -Tenodesis of *extensor carpi radialis*  -Arthrodesis of wrist  - Tenotomy of *Biceps, pectoralis major* | -T score after 3 months reached 52.29 from 38.50  -GAS scores improvement:   - 1.27 for Hygiene - 1.06 for pain - 1.00 for appearance - -9 hands with hypertonic deformity needed at least 1 additional BTX injection after surgery (mean; 10.4 after surgery) |
| **Tendon Transfer** | | | | | | |
| Keenan et al. 1987 (38) | **V** | Case  series | 34 | - Mean of 50 months | -FDS to FDP tendons transfer en masse  -Ulnar neurectomy distal to Guyon canal in 25 hands | -Pre-operatively: clench fist deformity with hygienic problems and no function in all patients  -Post-operatively: Open position in all hands which allowed for good hygiene of palmar surface |
| Peraut 2018 (41) | **V** | Case  series | 26 | - Mean of 47 months | **-**FDS to FDP tendons transfer en masse  -Wrist stabilization in 20 patients with wrist flexion> 20°  -Additional procedures if necessary: Ulnar neurectomy, wrist flexors tenotomy, procedures for thumb in palm | -Improvement in hand position 39from class V in all patients to:   - Class I (n=10) - Class II (n=12) - Class III (n=3) - Class IV (n=1)   -Improvement in House score from 0 to 0.88  -25 patents had no hygienic problems  - 7 patients had functional improvement |
| Facca et al. 2010 (39) | **V** | Case  Series | 19 | Mean of 6 months | - FDS to FDP tendons transfer en masse  -Additional procedures as required: ulnar neurectomy, tenotomy of wrist flexors/ *flexor policies longus,* tenodesis of wrist extensors | -MHS improved from 13.87 to 9.67 out of 20  -3 types of imperfect result:   - 1 insufficient opening of the thumb - 2 spasticity of intrinsic muscles - 1 wrist hyperextension |
| Heinen et al. 2008  (40) | **V** | Case  series | 6 | 19 months | - FDS to FDP tendons transfer en masse | -Hygienic problems resolved in 100% of cases  -Pain decreased in 5 cases  - Hand open position in all patients  -Passive extension of hand and wrist to neutral position in all cases  -All cases would agree to have the surgery one more time |
| Pomerance Keenan 1996 (55) | **V** | Case  series | 15 | 12months | A combination of STP, wrist arthrodesis, carpal tunnel release, FPL lengthening, wrist flexors release, ulnar neurectomy | -Hygiene problems resolved in all cases  -Improvement of hand/wrist position in all patients  -All caregivers reported improvements in appearance, condition to carry out hygiene activities, dressing |
| Pinzur et al. 1988 (42) | **V** | Case  series | 4 | From 26 to 36 | -*Brachioradialis* to *extensor digitorum communis* transfer | -Improvement in motor grade from  2 to 3  - Improvement in functional level from  1 to 4  -Improvement from no meaningful hand function to good assistive prehensile function |
| **Neurectomy** | | | | | | |
| Keenan et al. 1987(57) | **V** | Case  series | -21 cases of phenol injection  -21 cases of ulnar neurectomy | -25.8 months  In phenol  group  -24.3 months in neurectomy  group | -Phenol injection  -Ulnar neurectomy | -Phenol group: spasticity returned in  13 patients in 6 months follow up / 8 patents had no or little return  -Hand function improved in 6 patients  in phenol group and in 1 patient in neurectomy group  - Hygiene improved in all, except one case of neurectomy |
| Fouad 2011 (44) | **V** | Case  series | 10 | Mean of 21 months | Median and ulnar nerves selective neurectomy | - Recurrence due to insufficient sectioning reported in 1 case(10%)  -40% had more than 3 grades improvement of MAS  -40% had 2 grades of improvement of MAS  - 10% had one grade improvement of MAS  -10% reported no improvement of MAS  - Abnormal hand posture improved in 90% of patients  - 50% of patents had pain preoperatively which improved in all of them  - Overall, excellent results in 40%, good results in 40%, Fair results in 10% and poor results in 10% of patients |
| Maarrawi et al. 2006 (33) | **V** | Case  series | Total of 64 neurectomies in 31 patients,  Musculocutaneous (15), Median (25) and Ulnar (24) | 2, 6, 12 months and long term follow up of 4.5 years | Neurectomy | -AS improvement of forearm pronation, wrist and finger flexion and thumb adduction  -Spasticity decrease in the proximal muscles of the elbow (distant effect)  -Thumb in palm deformity decrease in  6 out of 10  -Significant improvement in resting joint position, active amplitude and motor strength of distal joints  -Patients’ mean satisfaction=61.5±24.6/ 100  -Family or nurse satisfaction = 64.15±19.47  - Improvement in functional and comfort goals |
| Sitthinam-suwan et al. 2013 (25) | **V** | Case  series | 33 medians  24 ulnars  Out of 141 total neurectomy | 21.1 months | -Neurectomy of median  motor branches *to pronator teres*, FCR, FDS  -Neurectomy of ulnar motor branch to FCU and 3th/4th FDP | -Median neurectomy:   - MAS improvement: 2.3 - PROM improvement: 18.9°   -Ulnar neurectomy:   - MAS improvement: 2.1 - PROM improvement: 21.3° |
| Pappas et al. 2010 (45) | **III** | Case  control | Group1:11  Group2:12 | 16.1 months | -Group1:STP, ulnar neurectomy, wrist  arthrodesis  -Group2: above procedures and median nerve recurrent branch neurectomy | -Group1: 5 patients developed an intrinsic TIP deformity/ hygiene problem resolved in 8 cases  -Group2: 2 patients developed an intrinsic TIP deformity/ hygiene problem resolved in 10 cases |
| **Arthrodesis** | | | | | | |
| Van Heest Strothman  2009 (47) | V | Case  series | 41 | after | -Wrist arthrodesis using  dorsal plating approach | - Disability assessment scale  improved from 9.6 to 5.5  - Appearance improvement (VAS= 7)  - Function improvement (VAS=6.0)  -Ease of daily care improvement (VAS=7.0)  -Hygiene improvement (VAS=6.2)  -94% of patients were satisfied  - Average satisfaction VAS score of 8.3  -Union rate(46) = 98%  -Four fractures through screw hole and 1 non-union  - Plate irritation, required hardware removal after union in 18 cases |
| Hargreaves  et al. 2000  (48) | **V** | Case  series | 11 | From 6 to 121 months | Wrist arthrodesis and additional procedures if necessary, including: FCU tenotomy, flexor aponeurotic release, STP transfer | -10 showed cosmetic improvement  -8 showed functional improvement  -Complications in 3 cases including:  loosening of K-wire, plate loosening, fatigue fracture of plate |
| Neuhaus  et al.2015  (56) | **V** | Case  series | 11 | 14 months | Wrist arthrodesis | -Improvement in mean radiographic flexion deformity from 65°to 4° dorsal angulation  -Improvement in House score in all patients with average of 2 levels  -Improved appearance in all cases |
| Rayan  Young  1999 (58) | **V** | Case  series | 11 | 32 months | Wrist arthrodesis | -Satisfaction of all patients  -Hygiene improved in all patients  -No skin palmar maceration or breakdown were reported  -All cases reported an improvement in appearance  -The average position of wrist fusion was 15° flexion  - The average amount of correction was 85° |
| **Thumb-in-palm deformity** | | | | | | |
| Botte  et al. 1989  (50) | **V** | Case  series | 27 | 39 months | -*FPL* tendon lengthening, Z-lengthening and fractional lengthening at the musculotendinous junction  -Release of first dorsal interosseous  -Arthrodesis of the thumb interphalangeal joint  - Z- plasty of the thumb web space were applied | -Improvement of hygiene care  -Satisfactory correction in 23 patients  -Functional improvements in all  patients who had volitional control before operation  -Pulp-to-pulp pinch restoration and useful grasp in 3 patients with arthrodesis |
| Goldner  et al.  1990 (59) | **V** | Case  series | -Total of 90  -Adult=22  -Children=68 | 6.5 years | -Only Arthrodesis (n=4)  -Arthrodesis, intrinsic muscle lengthening, tendon lengthening and/or transfer around wrist(n=4)  -MCP fusion, intrinsic muscle/tendon lengthening, extrinsic tendon plication, reinforcement/lengthening of the thumb flexor tendons | -Functional improvement in 84 out of 90  -Successful MCP joint arthrodesis in 86  -MCP joint solid fusion in 18 out of 22 |
| Smith  1982 (51) | **V** | Case  series | 7 |  | -FPL tendon transferring to the radial side of proximal phalanx of the thumb  -IP joint stabilization by tenodesis or arthrodesis (in 15° flexion) | -Appearance improved in all patients  -Thumb was not held in palm post operatively  -Increase in ability to use the hand for assistive grasp  -No improvement in manipulation of small objects and pinch |
| Pappas  et al. 2010  (43) | **III** | Case control | Group 1=11  Group2= 12 | 16.1 months | In addition to STP:  - Group1: ulnar neurectomy, wrist arthrodesis  -Group2: additional median recurrent neurectomy | -Group1: 5 patients developed thumb  in palm deformity  -Group 2: 2 patients developed thumb in palm deformity  - Hygiene related problems resolved in 8 out of 11 in group 1 and 10 out of 12 in group 2 |

AS: Ashworth Scale/ BTX: Botox/ COPM: Canadian Occupational Performance Measure/ FCR: *Flexor Carpi Radialis*/ FCU: *Flexor Carpi Ulnaris*/ FDS: *Flexor Digitorum Superficialis*/ FDP: *Flexor Digitorum Profundus*/ FPL: *Flexor Policis Longus*/GAS: Goal attainment scale / IP: Inter phalangeal/ MCP: metacarpophalangeal / MHS: Mini Hand Score/ STP: superficialis to profundus tendon transfer

**Figure 1: Data collection flowchart**

Spasticity & Surgery: 2347

- Medline: 981
- EMBASE: 1006
- CINAHL: 265
- Cochrane Central Register of Controlled Trials: 92
- Cochrane Database of Systematic Review: 3

CP: 1228

- Medline: 50
- EMBASE: 637
- CINAHL: 202
- Cochrane Central Register of Controlled Trials: 66
- Cochrane Database of Systematic Review:0

2 systematic reviews

Removed duplicated articles

ved duplicated articles

Spasticity & Surgery: 1802

- Medline: 977
- EMBASE: 655
- CINAHL: 109
- Cochrane Central Register of Controlled Trials: 60
- Cochrane Database of Systematic Review:1

CP articles:1053

- Medline: 505
- EMBASE: 390
- CINAHL: 99
- Cochrane Central Register of Controlled Trials: 59
- Cochrane Database of Systematic Review:0

2855 articles screened based on title and abstract

2015 articles excluded based on title and abstract

- Exclusion participants criteria
- Exclusion intervention criteria
- Exclusion language criteria

105 articles assessed for eligibility

25 full text articles excluded

- Exclusion participants criteria
- Exclusion intervention criteria

80 articles included in review

40 non trial articles

38 trial articles

**Appendix 1:**

**MEDLINE search strategy**

| 1 | exp Muscle Spasticity/ | 9126 |
| --- | --- | --- |
| 2 | exp Paraparesis, Spastic/ | 423 |
| 3 | exp Spastic Paraplegia, Hereditary/ | 1337 |
| 4 | exp Paraparesis, Tropical Spastic/ | 1649 |
| 5 | exp Cerebral Palsy/ | 20535 |
| 6 | (spastic* or cerebral pals*).ti,kw. | 25235 |
| 7 | (spastic* or cerebral pals*).ab. /freq=2 | 14534 |
| 8 | or/1-7 | 38474 |
| 9 | exp Muscle Spasticity/su or exp Paraparesis, Spastic/su or exp Spastic Paraplegia, Hereditary/su or exp Paraparesis, Tropical Spastic/su or exp Cerebral Palsy/su | 1935 |
| 10 | Surgical Procedures, Operative/ | 54884 |
| 11 | Orthopedic Procedures/ | 25602 |
| 12 | exp Tenotomy/ | 852 |
| 13 | exp Tendons/su | 13149 |
| 14 | exp Axotomy/ or exp Cordotomy/ or exp Rhizotomy/ | 4444 |
| 15 | Neurosurgical Procedures/ | 30402 |
| 16 | Reconstructive Surgical Procedures/ | 50017 |
| 17 | exp Arthrodesis/ or exp Bone Lengthening/ or Bone Transplantation/ or exp Diskectomy/ or Osteotomy/ or exp Tendon Transfer/ or exp Traction/ | 107436 |
| 18 | exp Nerve Transfer/ | 1963 |
| 19 | exp Lower Extremity Deformities, Congenital/su | 3080 |
| 20 | exp Upper Extremity Deformities, Congenital/su | 626 |
| 21 | exp Hand Deformities/su | 1892 |
| 22 | Foot Deformities/su | 591 |
| 23 | Foot Deformities, Acquired/su | 1284 |
| 24 | exp Tissue Expansion/ | 2337 |
| 25 | exp Joint Deformities, Acquired/su | 753 |
| 26 | (surgery or surgical* or operative* or operation* or resection* or neurosurg* or neuro-surg*).ti,kw. | 818373 |
| 27 | (surgery or surgical* or operative* or operation* or resection* or neurosurg* or neuro-surg*).ab. /freq=2 | 1032120 |
| 28 | (tenotom* or tendon release* or tendon lengthening* or heel-cord release* or tendon transfer*).ti,ab,kw. | 5352 |
| 29 | (neurectom* or neurotom* or nerve exeresis or nerve excision).ti,ab,kw. | 3081 |
| 30 | (arthrodes* or spinal fusion* or spine fusion or spondylodes* or spondylosyndes* or bone lengthening* or iliazarov technique* or iliazarov method* or distraction osteogenes* or callotas* or limb lengthening* or bone transplant* or bone graft* or bone autograft* or bone flap* or osseous flap* or osteoarticular graft* or bone allograft* or fibula* graft* or fibula* autograft* or fibula* flap* or fibula* transplant* or fibula* osteocutaneous flap* or diskectom* or discectom* or percutaneous nucleotom* or osteotom* or traction* or nerve transfer* or neurotization* or nerve crossover* or neuroanastomos* or nerve allotransplant* or nerve graft* or nerve tissue transplant* or nerve transfer* or nervous tissue transplant* or tissue expansion* or nerve expansion* or nerve stretching* or nerve elongation* or nerve lengthening* or neurolysis or neuronolysis or neurotom* or nerve transection* or nerve transsection* or axotom* or axon transection* or axon transsection* or axonotom* or nerve fibre section* or nerve fiber transection* or nerve fiber transsection* or nerve fibre section* or nerve fibre transection* or nerve fibre transsection* or rhizotom* or thermorhizotom* or spinal cord decompression* or spinal decompression* or bone reimplant* or capsul* release or hip reconstruction or shoulder reconstruction or fasciculotom* or cryoneurolysis).ti,ab,kw. | 132320 |
| 31 | or/9-30 | 1668605 |
| 32 | 8 and 31 | 4440 |
| 33 | baclofen.ti. | 2776 |
| 34 | (surgery or surgical* or operative* or operation* or resection* or neurosurg* or neuro-surg* or tenotom* or tendon release* or tendon lengthening* or heel-cord release* or tendon transfer* or neurectom* or neurotom* or nerve exeresis or nerve excision or arthrodes* or spinal fusion* or spine fusion or spondylodes* or spondylosyndes* or bone lengthening* or iliazarov technique* or iliazarov method* or distraction osteogenes* or callotas* or limb lengthening* or transplant* or graft* or autograft* or bone flap* or osseous flap* or allograft* or fibula* flap* or fibula* osteocutaneous flap* or diskectom* or discectom* or percutaneous nucleotom* or osteotom* or traction* or nerve transfer* or neurotization* or nerve crossover* or neuroanastomos* or allotransplant* or nerve transfer* or tissue expansion* or nerve expansion* or nerve stretching* or nerve elongation* or nerve lengthening* or neurolysis or neuronolysis or neurotom* or transection* or transsection* or axotom* or axonotom* or nerve fibre section* or nerve fibre section* or rhizotom* or thermorhizotom* or spinal cord decompression* or spinal decompression* or bone reimplant* or capsul* release or hip reconstruction or shoulder reconstruction or fasciculotom* or cryoneurolysis).ti. | 1201376 |
| 35 | 33 not 34 | 2734 |
| 36 | 32 not 35 | 4362 |
| 37 | limit 36 to (english or french or persian) | 3748 |
| 38 | exp Adult/ | 7176435 |
| 39 | 37 and 38 | 1134 |
| 40 | exp Infant/ or exp Child/ or exp Adolescent/ | 3547810 |
| 41 | 37 not 40 | 1471 |
| 42 | 39 or 41 | 2174 |
| 43 | (adult* or matern*).ti,jw. | 456177 |
| 44 | 42 and 43 | 93 |
| 45 | (child* or adolescen* or pediatr* or paediatr* or infant* or newborn* or neonat* or fetus or fetal).ti,jw. | 1816971 |
| 46 | 42 not 45 | 1587 |
| 47 | 44 or 46 | 1609 |
| 48 | (exp Animals/ not exp Humans/) or exp Mice, Transgenic/ or exp Transgenes/ or exp Models, Animal/ or veterinary.fs. or exp Xenograft Model Antitumor Assays/ or veterinar*.jw. | 5050002 |
| 49 | 47 not 48 | 1565 |
| 50 | (Address or Biography or Comment or Congress or Editorial or Ephemera or Introductory Journal Article or News or Newspaper Article or Popular Work or Webcast or lectures or sermons or blogs or interviews or personal narratives or autobiography or overall or advertisements or letter or posters or programs or prospectuses or juvenile literature or patient education handout).pt. | 2325917 |
| 51 | 49 not 50 | 1515 |
